# Discovering activity transition patterns in social media check-in behavior via temporal activity motifs

**DOI:** 10.1038/s41598-025-14843-x

**Published:** 2025-08-08

**Authors:** Rui Zhao, Yong Gao

**Affiliations:** Institute of Remote Sensing and Geographic Information Systems, School of Earth and Space Sciences, Peking University, Beijing, China

**Keywords:** Location-based social network, Check-in data, Activity sequence, Activity type transition, Temporal motif, Temporal activity motif, Psychology and behaviour, Complex networks, Scientific data

## Abstract

Location-Based Social Network (LBSN) has produced a large quantity of user check-in data. A profound understanding of user behavior and intrinsic needs can be achieved by identifying patterns in activity type transitions, thereby enabling more intelligent location-based services. We proposed temporal activity motif and used this structure to identify frequent activity type transition patterns from check-in sequences, discovering the relationship and interaction between different activity types. 383 temporal activity motifs of 17 temporal topologies were extracted from the two-year Gowalla dataset of New York-Newark-Jersey City, NY-NJ-PA Metropolitan Statistical Area (MSA). These motifs are categorized into two groups: one is sequential motifs representing a complete activity process, while the other is non-sequential motifs representing the co-occurrence of two separate processes. They provide evidence of activity type recurrence, particularly in longer activity processes, highlights the cyclical nature of human mobility. Additionally, various activity types exhibit different influences on others and occupy different positions in activity processes. Furthermore, by leveraging non-sequential motifs, we specifically uncovered the co-occurrence patterns between two separate activity process. These findings bring new insights to optimize recommendation system and urban planning.

**Supplementary Information:**

The online version contains supplementary material available at 10.1038/s41598-025-14843-x.

## Introduction

Mobile phones have become indispensable tools in our daily lives. People can not only obtain location-based services, such as searches, navigation, and recommendations, but also produce location-tagged data by checking in, sharing, and posting. This enables platforms to collect plenty of semantically rich user activity data, facilitating the development of personalized and intelligent services^1,2^. Under the guidance of social medias^3^, people are used to check in at places where they carry out activities, which helps to form a sustainable data flow^4,5^. Therefore, compared with other types of social media data, check-in data can more comprehensively reflect continuous user activities. With the popularity of location-based social network (LBSN) such as Gowalla and Foursquare, check-in data incorporates additional information beyond location, such as POI (Point of Interest) and category details, providing foundational data support for exploring activity patterns in people’s travel behavior^6–9^.

Compared to specific POIs or locations where activity occur, activity types more directly reflect people’s daily demands, offering a higher-level and semantically meaningful abstraction of user behavior. By focusing on activity types, we aim to uncover generalizable patterns of human demand and behavior that are not limited by spatial granularity and urban layout. Furthermore, transitions between different activity types reflect dynamic changes of people’s needs. Particularly, higher-order transitions can reveal complex demand structures and activity patterns.

Conventional research primarily relied on detailed survey data, often incorporating sociodemographic characteristics into the analysis^10,11^. In recent years, however, the widespread availability of mobile phone and social media data has enabled the discovery of more prevalent and diverse patterns from vast datasets. Researchers have investigated the temporal distributions^1,6,10–13^, spatial distributions^4^, and temporal interval distributions^13,14^ of various activity types. Among these, different temporal scales have garnered particular attention^12^. Conversely, for activity data without behavioral semantics, spatiotemporal patterns and duration times can be leveraged to infer potential activity types^15^. Researchers also found frequent transitions between specific activity types^6^, and used transition probability matrix to describe this phenomenon^1,4,14^. At the urban scale, transition patterns between specific zones are also used to infer their functional semantics^16^. Moreover, users’ interests and preferences for specific activity types reflect their personalized lifestyles or specific societal roles^17,18^. Using methods such as k-means clustering^1^ and hierarchical clustering^11^, researchers successfully identified distinct groups of people, including office workers, homebodies, and students. They also found that people with similar work domains or preferences for visiting the same communities are more likely to exhibit similar daily activity patterns^17,19^. Incorporating contextual features of activity type patterns^6,14,20^ and the predictability of activity transitions^21^ into the model enhanced the accuracy and user experience of recommendation or prediction algorithms. However, earlier studies primarily focused on the distribution and pattern of every single activity type. Apart from transitions between two activity types, they rarely paid attention to the relationships among multiple types.

A structure called motif is well-suited for studying higher-order activity type transitions. Motif first appeared in the field of complex networks. Milo et al. defined it as subgraphs that frequently appear in networks^22^. By analogy, Schneider et al. proposed location-based individual mobility motifs^23^, where vertices represent locations and edges represent user movements, to analyze user mobility patterns. Jiang et al. viewed human behavior as an organization of a series of activities, which is highly structured, making it suitable for abstraction and representation through motifs^24^. Yang et al. proposed semantic travel motifs, and explored user preferences by calculating the priority score of each semantic type^25^. Cao et al. proposed activity-based motifs^26^, where vertices represent activity types and edges represent transitions between them, to model transition patterns. However, due to limitations in call detailed records (CDR), only home and work can be identified, while the remaining types are categorized as social activities. Although call detailed records can accurately capture users’ location and stay time, it naturally lacks semantic information about what users are doing^1^. Yin and Chi discovered richer activity patterns by analyzing activity-based motifs with 11 activity types inferred from land use data^27^. Lei et al. proposed temporal motifs, where edges are timestamped to preserve the sequential order of user movements^28^. This facilitated the observation of dependencies and causal relationship in multiple activity type transitions. Unfortunately, the last two studies didn’t further explore the regularities underlying activity type transitions. Additionally, they relied on land use data to infer activity type, which is not reasonable enough since multiple activities can occur simultaneously within the same land use type^4,29^.

In this study, we proposed temporal activity motifs to represent patterns of activity type transitions. In this framework, nodes represent activity types, and timestamped edges represent ordered transitions between different types. With the clarification of transition order, we can clearly understand the roles and positions of various activity types within activity processes. We adapted and extended the temporal motif counting method proposed by Paranjape et al.^30^ to develop an identifying method for temporal activity motifs. This method is computationally efficient, making it possible to participate in real-time POI recommendations. On this basis, we not only identified low-order, sequential motifs but also high-order, non-sequential motifs from check-in data. Non-sequential motifs refer to those whose edges are not entirely sequentially connected, representing the co-occurrence of two separate activity processes and revealing associations between them. These motifs are prevalent but always neglected in traditional motif research. We aim to uncover the underlying characteristics and patterns them exhibit. By using user check-in data, we obtained activity type sequences that closely reflect real-world user behaviors. Additionally, the POI category information embedded in check-in data allows us to infer activity types naturally and accurately^4,7^.

## Analysis of temporal activity motifs

We utilize a check-in dataset originally collected from Gowalla (https://www.gowalla.com/), a location-based social networking website. The dataset covers the New York-Newark-Jersey City, NY-NJ-PA Metropolitan Statistical Area (MSA), spanning from March 2009 to July 2011, and includes check-in records from 20,944 users across 50,790 Points of Interest (POIs). See the Methods section for further details. Using this dataset, we extracted users’ daily activity type sequences and identified 383 temporal activity motifs. These motifs reveal local activity transition patterns within users’ daily routines.

**Fig. 1:**
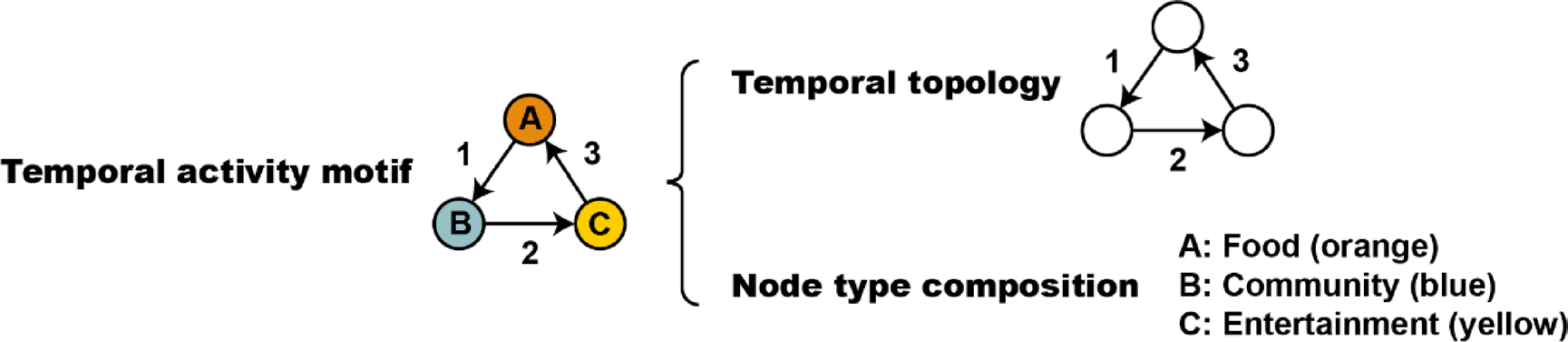
Temporal activity motif and its two key features.

The detailed structural description of the temporal activity motif is shown in Fig. 1. Temporal activity motif is characterized by two key features: temporal topology and node type composition. Temporal topology refers to the graph’s shape, edge direction and edge order, which determines the specific transition pattern. Node type composition refers to the activity types corresponding to each node in the transition pattern. In our study, all motifs are extracted from activity transitions occurring within a single day, as users’ activity type sequences are segmented into daily intervals. This imposes an implicit 24-hour temporal boundary on each motif, ensuring they reflect intra-day behavioral patterns aligned with human circadian rhythms. Temporal activity motifs focus on activity type transitions and do not incorporate spatial information.

### Temporal topologies of temporal activity motifs

**Fig. 2:**
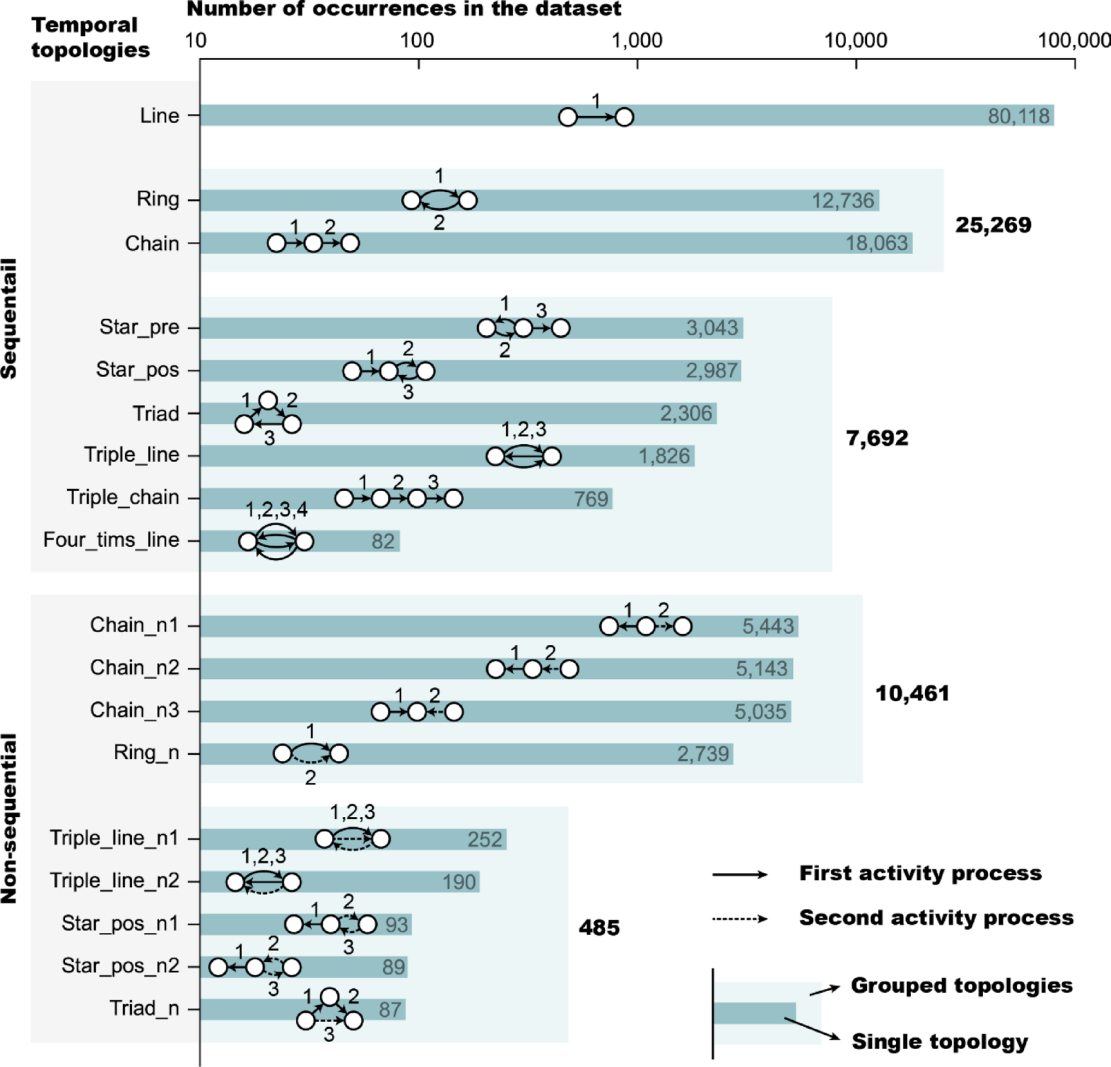
The schematic diagrams of 17 temporal topologies and the number of their occurrences in the dataset (i.e., the number of activity sequences containing them).

In this section, we analyze the quantitative distribution and representativeness of motifs categorized by temporal topologies and uncover the phenomenon of activity type recurrence. The identified motifs belong to 17 different temporal topological structures, with their schematic diagrams and the number of their occurrences in the dataset presented in Fig. 2. The number of occurrences (i.e., topology occurrence) refers to the total count of instances in which these topologies appear, with details in the Methods section. The topologies and their contained motifs are classified into two categories: sequential and non-sequential. Sequential motifs represent complete activity processes, with 204 identified motifs across temporal topologies including Line, Ring, Chain, Triple_line, Four_times_line, Star_pre, Star_pos, Triad and Triple_chain. Non-sequential motifs represent two or more discontinuous processes, with 179 identified motifs across temporal topologies including Ring_n, Chain_n1, Chain_n2, Chain_n3, Triple_line_n1, Triple_line_n2, Star_pos_n1, Star_pos_n2, and Triad_n.

The Motif Coverage Ratio (MCR) was computed by group to quantify how well the topologies account for the dataset. It is defined as the ratio of topology occurrence to the number of daily activity sequences that structurally allow the contained motifs, thereby providing a measure of their representativeness for activity type transition patterns. See the Methods section for a detailed explanation.

In sequential motifs, Line represents single transition, and every transition in user activity sequence can be identified as a Line motif. With an MCR up to 100%, Line motifs reveal simple but universal rules, playing a significant role in activity inference and overall trend analysis. Ring and Chain motifs represent two consecutive activity type transitions, with an MCR of 71%. The rest are higher-order motifs, with an MCR of 45%. In the process of candidate pattern counting, 6235 sequential high-order patterns from 19 temporal topologies were involved, but only 100 motifs from 6 temporal topologies were identified as high-frequency motifs. Less than 2% patterns reach an MCR of 45%, which means a few motifs can greatly enhance our comprehension of users’ complex activity behavior.

In non-sequential motifs, edges are not connected in temporal order. Particularly, two-edge non-sequential motifs have an MCR up to 61%. Non-sequential motifs have never gained enough attention in previous studies, but they are conspicuous and exist in many users’ activity sequences. Rather than representing a complete transition process, they reflect the co-occurrence between two separate processes, which inspire us about the relationship between prior and posterior transitions.

Many studies on human mobility have demonstrated people’s preference to revisit their favorite places. Similarly, analysis of sequential motifs reveals that people also tend to repeat the same activity types in their daily schedule, especially when they have longer activity sequences. In sequential temporal topologies, some are chain-like, while others contain cycles. The existence of a cycle indicates that a user repeats the same type of activity in a complete sequence. We named this phenomenon activity type recurrence. In two-edge motifs, non-cycle motifs occur more frequently. However, the situation is different for three-edge motifs, where motifs with cycles become more prevalent. The more activities users participate in, the more likely they are to repeat previous activities. Activity type recurrences also exist in non-sequential motifs. In every non-sequential topology, the two activity processes share at least one node, indicating that the activity type performed earlier is repeated in the later process.

### Sequential motifs: patterns within a single activity process

By analyzing node type compositions and activity type distributions in different temporal topologies, we identified some typical user behaviors, and revealed the roles of different activity types within individuals’ daily routines. We firstly rely on the intuitive comparisons provided by activity type distribution visualizations to identify general trends. Moreover, we introduce the Cumulative Occurrence Ratio (COR) as a complementary metric rigorously assess the importance and involvement of motifs with specific semantics. COR is the ratio of the cumulative occurrences of the target motifs to the cumulative occurrences of all motifs, with details in Methods section.

#### Two-node sequential motifs

**Fig. 3:**
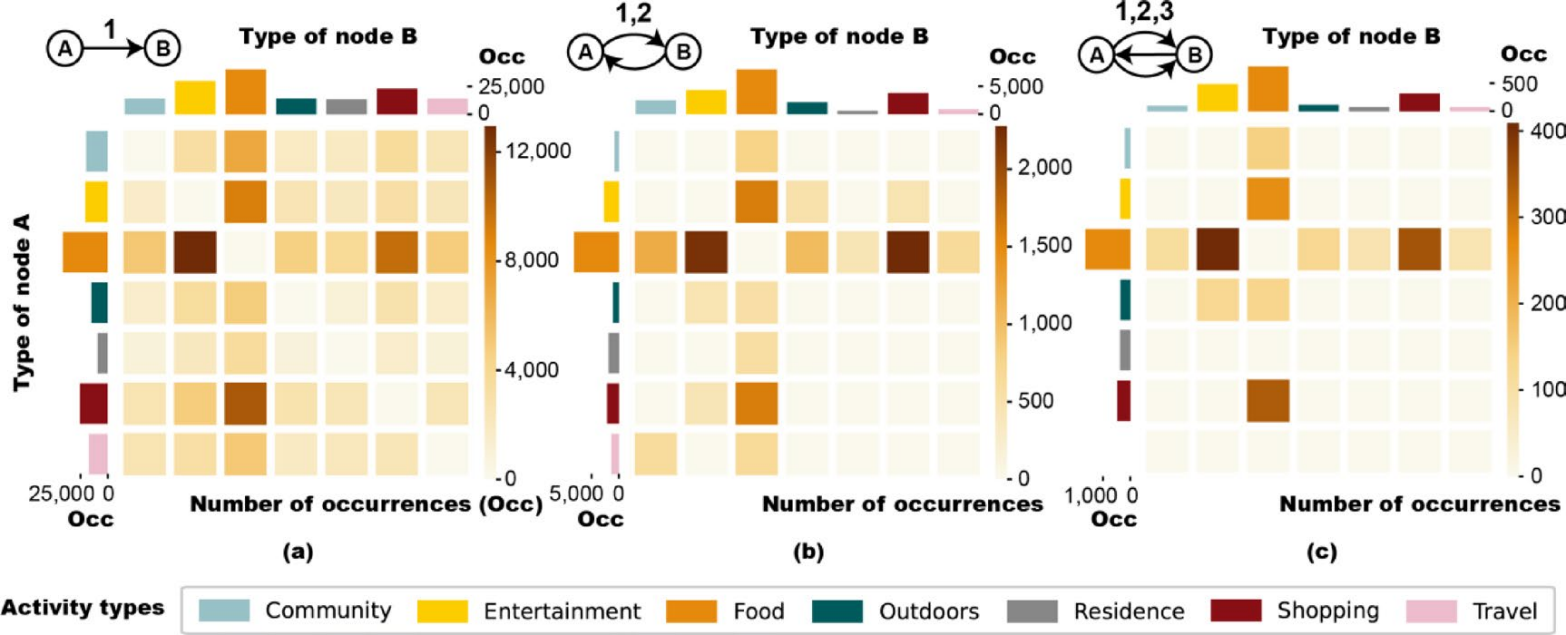
The activity type distributions in Line **(a)**, Ring **(b)**, and Triple_line **(c)** motifs. The distribution of activity type transitions is shown in the heat map, where rows represent types of node A and columns represent types of node B. The type distributions of each single node are shown in bar charts on the left side and top of the heat maps.

Line represents one-way transition between two types, where exists a push and pull that prompts users to change what they do. Figure 3a shows the activity type distributions in Line motifs. The diagonal symmetry in the heat map indicates that the driving force and attractions between various activity types are bidirectional and balanced. When transitions from one activity type to another are frequent, the reverse transitions are also common, and vice versa. The Food type participates in a majority of cases (COR = 55%), either driving people to or attracting them from other activities. The Entertainment and Shopping types, related to commercial activities, interact with Food frequently, which can be informative for commercial space planning. For example, in large shopping malls, a balanced arrangement of restaurants, shops, and entertainment venues can encourage consumers to stay longer and spend more money. Frequent transitions between Community and Entertainment/Shopping may suggests that people prefer to relax themselves after work to alleviate tension and stress. On the contrary, Residence rarely participates in Line motifs (COR = 15%). This may be related to the bias of check-in data, as users are more likely to check in during social activities than at home. Besides, home-based activities are highly enclosed, limiting people’s physical contact with outside world and reducing their willing to go out.

Ring represents cycle between two types. Usually, Ring cycles between core demand and supplementary demand. Sometimes, Ring also represents biological rhythms or reflect scenario limitations, where the second edge represents a return with constraints. Figure 3b shows the type distributions in Ring motifs. Transitions related to Food in Ring motifs are also common. Food is often the core need (COR = 49%), as it is one of the necessities of human beings and affects circadian rhythms. And it can also serve as a temporary or supplementary activity (COR = 35%), meeting needs such as eating, socializing, and business negotiations. To improve customer satisfaction, the arrangement of catering facilities can be optimized according to people’s main activities, needs and consumption levels. On the other hand, when users are carrying out other activities, the recommendation system can suggest nearby restaurants at contextually relevant times. There’re still some transitions unrelated to Food. The ones between Entertainment and Outdoors represent prolonged leisure time, combining outdoor and indoor activities. This suggests that indoor entertainment may benefit from complementary outdoor activities that help balance physical and mental well-being. Creating convenient transportation links between entertainment venues and nature attractions can make it more convenient for city dwellers to relax in their spare time. The ones between Entertainment and Shopping represent repeated commercial activities. The last one is Travel → Community → Travel, possibly representing business trips or commuting behaviors. The route between station and workplace is usually fixed and time-limited, resulting in a point-to-point travel mode.

Triple_line consists of three consecutive transitions between two activity types. Figure 3c shows the type distributions in Triple_line motifs. Food has frequent interactions with Entertainment or Shopping (COR = 63%), which means loops are common between dining and commercial activities. Besides, the relationship between Food and Entertainment is more asymmetric. People tend to engage in Food and then Entertainment (COR = 18%), rather than the reverse (COR = 12%).

#### Three-node sequential motifs

**Fig. 4:**
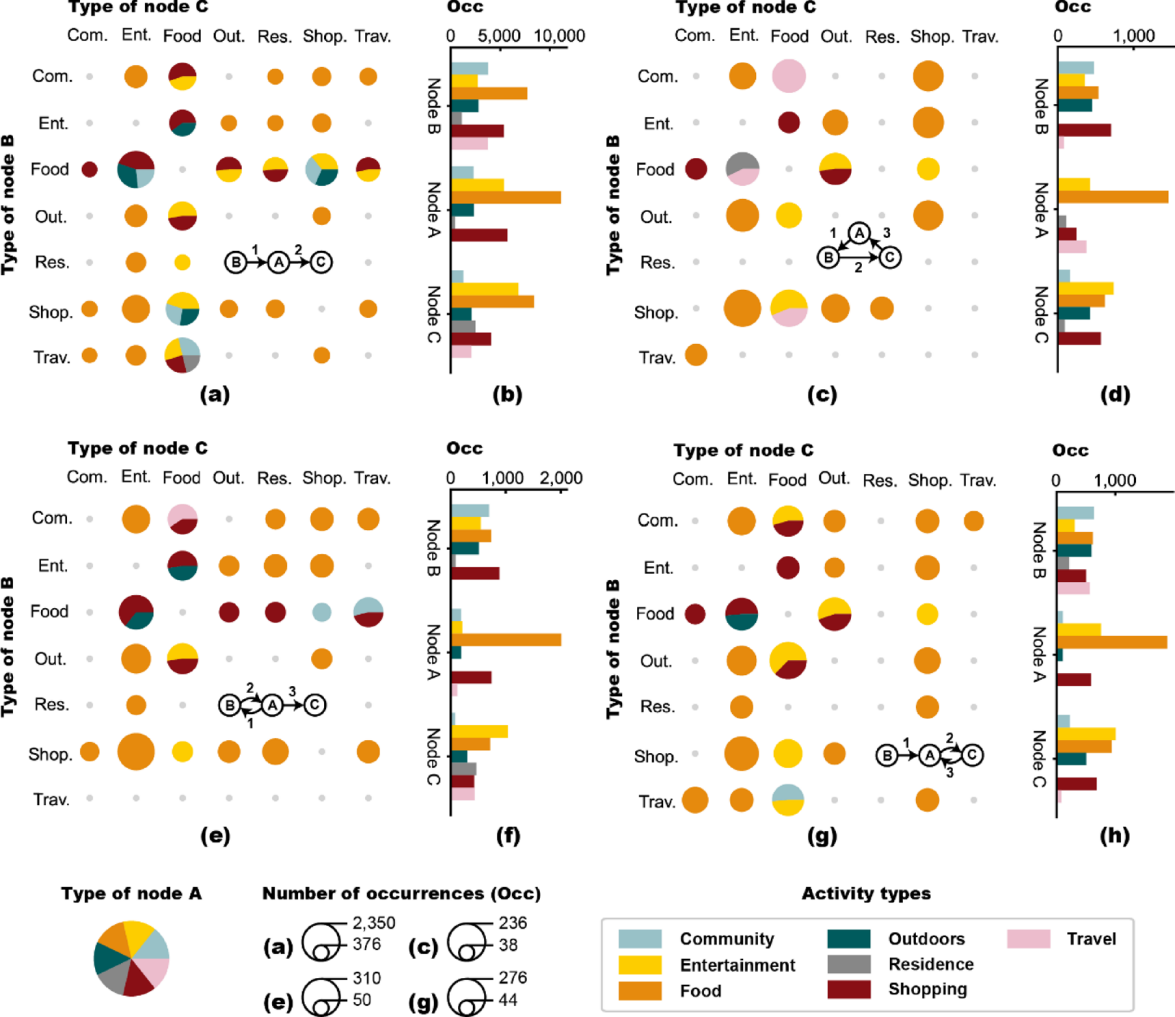
The activity type distributions in Chain **(a**,** b)**, Triad **(c**,** d)**, Star_pre **(e**,** f)**, and Star_pos **(g**,** h)** motifs. The distributions of activity type transitions are shown in a series of grouped pie charts in **(a**,** c**, **e**,** g)**, where rows represent types of node B, columns represent types of node C, colors of the pie represent types of node A, and pie sizes represent numbers of occurrence of specific motifs in dataset. The type distributions of nodes A to C are shown in bar charts in **(b**,** d**, **f**,** h)**.

Chain represents one-way transitions between three activity types. These activity types are logically coherent, which shows a natural extension of user demands. Sometimes, there’s an inevitable change driven by time constraints. Sometimes, the geographical proximity of different activity venues naturally leads to the grouping of certain activities. Researchers have presented the distribution of transitions on each temporal edge separately^28^. Since we want to observe the relation among temporal edges, we draw combined pie charts to show the type distribution throughout the activity process. Figure 4a, b show the activity type distributions in Chain motifs. We found that Food must be one of the three activities (COR = 100%). And one of Entertainment or Shopping always participates in Chain (COR = 91%), which fully demonstrates that people tend to integrate commercial activities. In addition, Travel → Food → Community and its reverse represent typical business trips or commuting patterns. Motifs involving both Travel and Food are often associated with visitors, who prefer to choose Shopping as the third activity type. Entertainment is more common in the latter part of the activity processes (COR = 45%) than at the beginning (COR = 10%), confirming its role as a natural extension of preceding activities that helps people relieve stress and relax. Adding self-service entertainment facilities around core activity venues provide convenience for consumers in need. The main function of Residence and Travel is to guide people into or out of the activity processes, so it is reasonable that they mostly appear at the beginning or the end (COR = 35%) rather than in the central position (COR = 2%). Community usually appears in the early stage (COR = 22%) rather than at the end (5%), indicating that it acts more as a driver than an attractor, initiating subsequent activities.

Both Star_pre and Star_pos consist of a cycle and a branch. In Star_pre, the cycle comes first and the branch follows, paying attention to the extension of core demands. While in Star_pos, the branch comes first and the cycle follows, attaching more importance to the activities before core demands. Figure 4e-h present the activity type distributions in Star_pre and Star_pos. The central node A represents the core demand, which has a stable attraction and connects other activities. In both Star_pre and Star_pos, Food is the most common central type (COR = 58% and 55%). Regular eating is the basis for good everyday life, so people eat at fixed times and arrange other activities in between, thus forming such patterns. Shopping is also common as a core type. Entertainment, however, is common only in Star_pos, indicating that people may pay more attention to preceding activities when Entertainment is the primary demand. The return node (B in Star_pre, C in Star_pos) aims to trigger the recurrence of the core demand. In Star_pre, the types of return nodes are evenly distributed. However, in Star_pos, Entertainment and Food are more prevalent (COR = 57%), while Community is less common (COR = 4%). This suggests that as a supplementary demand, Entertainment and Food are more concerned with preceding activities, while Community is the opposite. In Star_pre, node C is on the branch and at the end, representing the supplementary and final activities. Entertainment tends to act as the end (COR = 30%), as an effective way to free people from their busy day and gather energy for the day ahead. Residence and Travel are also more likely to occur at the end (COR = 26%) compared to other positions (COR = 6%), since users are more likely to check in when they get home or on their way home to finish a day. In Star_pos, node B is on the branch and at the beginning, representing the starting activities, prompting people to carry out preceding activities. Community and Travel tend to be the beginning here (COR = 35%), suggesting that both getting off work and arriving at a station can stimulate subsequent activities. For the benefit of the public, some new solutions in urban planning should be implemented, such as 15-minite neighborhood, transit-oriented development (TOD), and mixed-use development. Moreover, by integrating relaxing and commercial spaces into office environments, officers can get better relaxation and higher work efficiency.

Triad represents cycle among three activity types. Figure 4c, d present the type distributions of transitions and nodes in Triad motifs. In most cases, the central node A represents the core demand, which is stronger than those in Star_pre and Star_pos, as users return to the core need even after completing two supplementary activities. Food is still the most common central node (COR = 56%). However, in some cases, node A dose not signify a core requirement. For instance, the Travel type primarily appears as node A. When people travel from another city to the current city, or from the suburbs to the city center, the station where they arrive and leave is often the same, forming the cycle-like patterns. These people usually travel for business or shopping purposes, which serve as their core needs. Nodes B and C can be core travel purposes or supplementary needs. Entertainment, Food, Outdoors, and Shopping all participate in node B or C. Outdoors and Shopping appear more often in node B (COR = 38%), because these activities require a lot of energy, so people tend to do such activities at the early stages. Entertainment and Food appear more often in node C (COR = 34%), arranged at a later stage, since these activities can help people relax and eliminate fatigue. Community is more likely to appear in node B (COR = 18%) and is rarely observed in node C (COR = 6%), reaffirming its role as a pioneering activity.

**Fig. 5:**
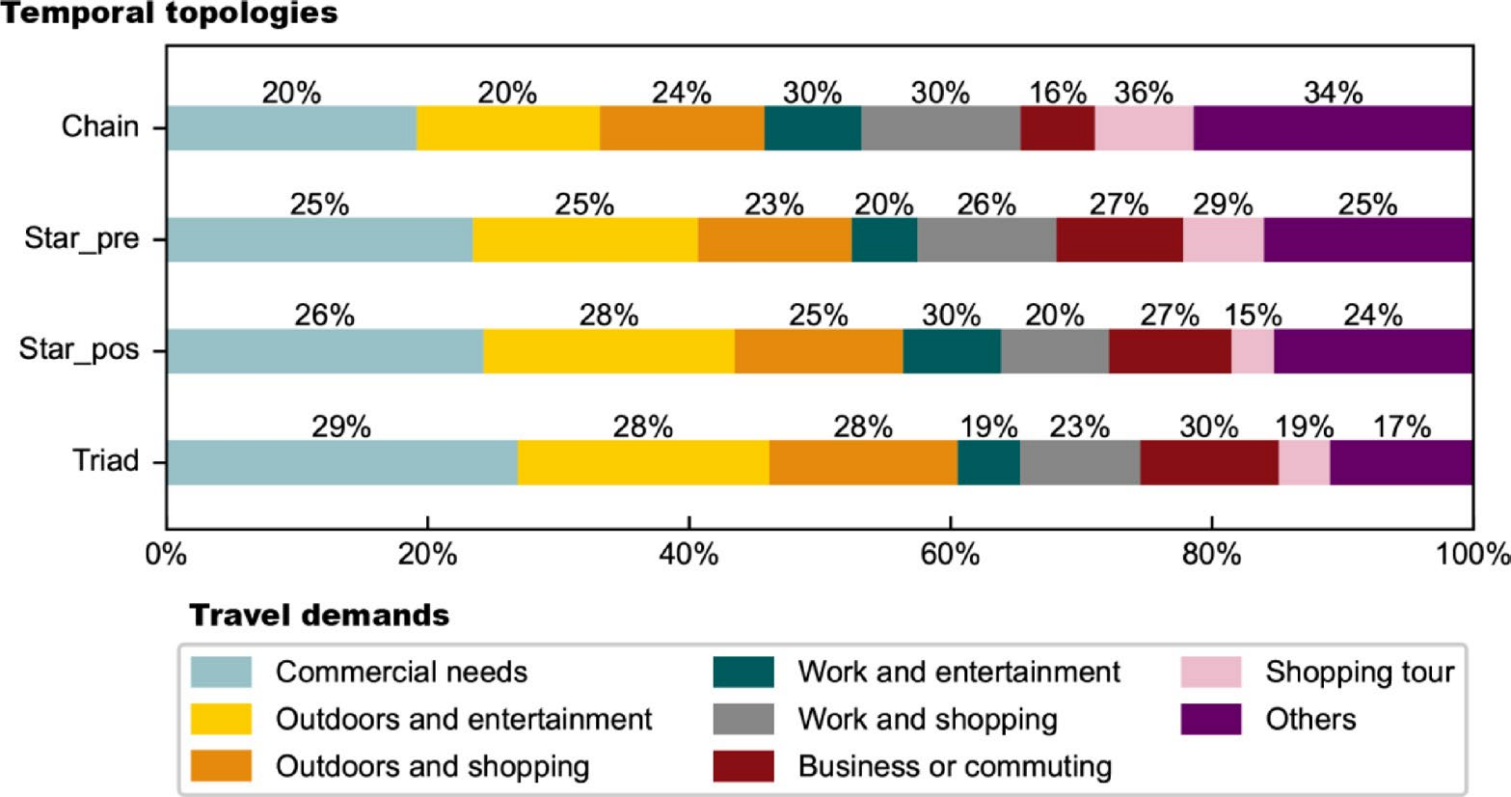
The Cumulative Occurrence Ratio of motifs with different typical travel demands in three-node temporal topologies (Chain, Star_pre, Star_pos, and Triad).

Some activity type combinations are frequently observed in Chain, Star_pre, Star_pos and Triad motifs. We selected seven typical user travel demands, each involving three activity types: Commercial needs (Entertainment, Food, Shopping), Outdoors and entertainment (Entertainment, Food, Outdoors), Outdoors and shopping (Food, Outdoors, Shopping), Work and entertainment (Community, Entertainment, Food), Work and shopping (Community, Food, Shopping), Business or commuting (Community, Food, Travel), and Shopping tour (Food, Shopping, Travel). Figure 5 presents the COR of motifs with different demands in the four three-node temporal topologies respectively. The results show that “commercial needs”, “outdoors and entertainment”, and “business or commuting” tend to appear in those motifs with cycles, indicating a strong tendency for activity recurrence, while “work and shopping” and “shopping trip” tend to appear in chain-like motifs. In addition, there are preferences for different temporal topologies. For example, “commercial needs”, “outdoors and shopping” and “outdoors and entertainment” show the preference for Triad, where eating is the essential need and leisure activities are as supplements. “Work and entertainment” tends to appear in Star_pos, where work is always the beginning of a series of activities. “Shopping trip” tends to appear in Star_pre, where stations are always the place to leave.

#### Four-node sequential motifs

**Fig. 6:**
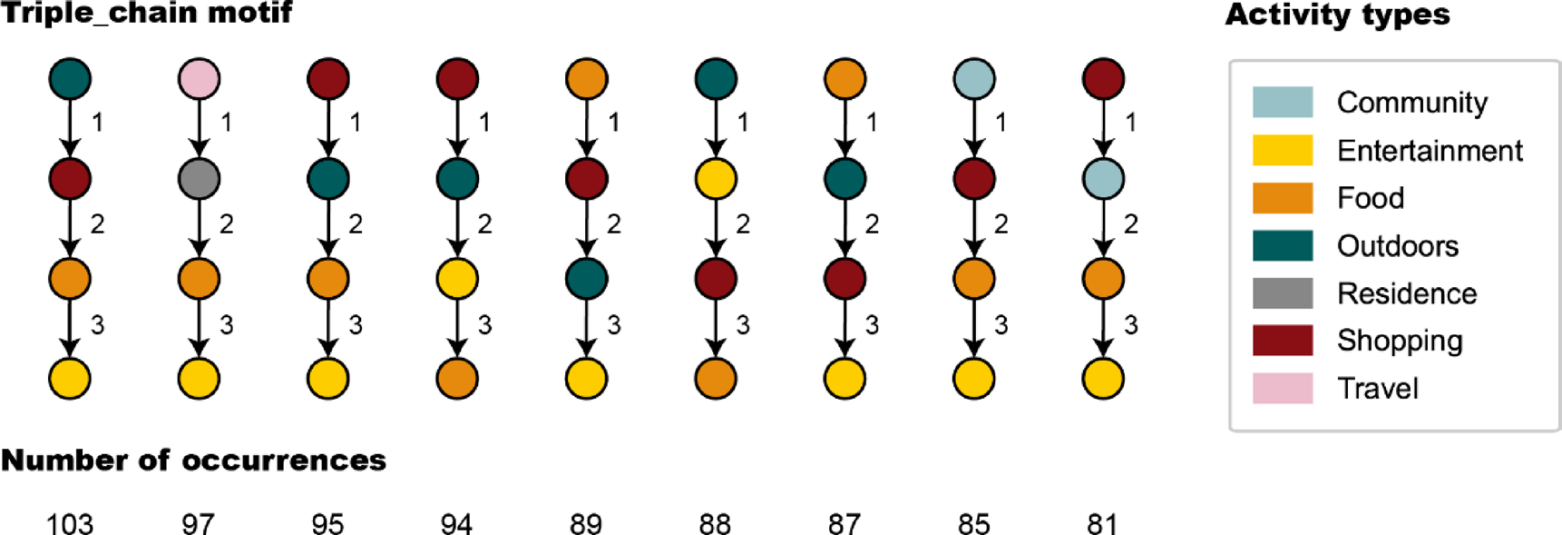
Schematic diagrams of nine Triple_chain motifs.

Triple_chain represents one-way transitions between four activity types. In the process of candidate pattern counting, 836 patterns of Triple_chain were considered, while only 9 of them were identified as motifs, presented in Fig. 6. Six of them are consist of Outdoors, Shopping, Food, and Entertainment (COR = 68%), which means there is a strong correlation and mutual attraction within these four types of activity. These may be the typical patterns people present on their days off, arranging various leisure activities to relax their body and mind. At the same time, the closer distribution of various functional spaces such as shopping mall, restaurants, entertainment venues and outdoor spaces also leads to these activity patterns. In addition, Entertainment typically appears at the end of activity processes (COR = 78%), while Shopping and Outdoors are more likely to appear at early stage (COR = 67% and 44%, respectively). The results show the inertia of human behavior, which is observed many times in the previous results. Besides, two of them describe a series of leisure activities after work, and the last one describes the process of arriving, checking in at the hotel, and sightseeing.

### Non-sequential motifs: co-occurrence patterns of different activity processes

**Fig. 7:**
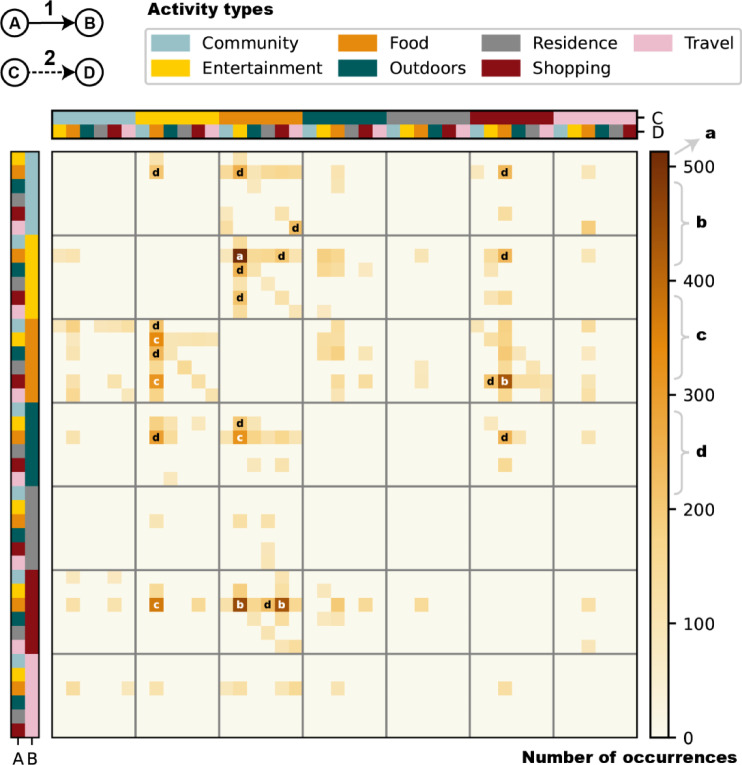
The activity type distribution of transitions of two-edge non-sequential motifs. High values are labeled with lowercase letters, with ‘a’ representing the number of occurrences greater than 500, ‘b’ between 400 and 500, ‘c’ between 300 and 400, and ‘d’ between 200 and 300. Each row represents a type of transition A→B, and each column represents a type of transition C→D. Node B is the end of the previous process, and node C is the beginning of the subsequent process. They form the bridging mode in non-sequential motifs. Motifs within the same large grid share a bridging pattern, meaning the types of B and C are identical.

Non-sequential motifs consist of edges not fully sequentially linked, representing two or more separate processes of activity type transitions. In fact, due to the differences in user personalization, there are some unvaluable activities in check-in data most of the time, preventing researchers from discovering the commonalities between different activity type sequences. Non-sequential motifs model the co-occurrence between various activity processes, ignoring differences in intermediate activities. Two-edge non-sequential motifs generally exist in the dataset, where the length of the two processed is 1, reflecting the correlation between the simplest transfers. Figure 7 illustrates the co-occurrence relationship between the previous and subsequent transitions. The heat map is divided into 49 large grids, each shares the same types of nodes B and C, representing the same bridging mode.

There are many high values in the heat map, indicating strong correlations between specific transitions. There are 8 extremely high values exceeding 300. Among them, four involve repeated identical processes. The repeated transitions are Entertainment → Food, Shopping → Food, and their reverses, all of which are transitions between catering and commercial activities. Additionally, three involve dining, shopping, and entertainment activities at the same time. Dining appears in both processes, shopping only appears previously, and entertainment only appears subsequently. The final one is Food → Outdoors … Food → Entertainment (“…” means a bridging pattern), an outdoor-to-indoor leisure pattern.

Hotspots exist in the heat map, which stretch horizontally or vertically. Horizontal aggregation indicates that specific initiating activities and bridging modes can trigger multiple activities, such as Food → Community/Entertainment/Outdoors/Shopping … Food. Vertical aggregation indicates that specific bridging modes and final activities can attract multiple preceding activities, such as Food … Entertainment/Shopping → Food. When high values gather into hotspots, commercial activities always participate in the bridging modes and play a hub role (COR = 96%). In addition, some high values are isolated, with specific start and end types and bridging modes, such as Food → Community … Entertainment → Food and Travel → Community … Food → Travel. Aggregated hotspots reveal structures with universal attractiveness or driving force, helping predict future user activity. However, isolated high values reveal special patterns, helping generate personalized user portraits. They can play complementary roles in recommendation systems.

Moreover, diagonal hotspots exist in some large grids, with the same node types of nodes A and D, forming a cycle in the end. This observation not only provides evidence of activity type recurrence in segmented activity processes but also suggests that complete activity processes tend to form closed loops, particularly through commercial bridging patterns like Food … Entertainment, Food … Shopping and their reverses. In recommendation systems, we can try to predict the revisit behavior over longer intervals by identifying specific bridging modes.

## Discussion

### Summary and insights

In this study, we proposed the definition of temporal activity motif, modeling higher-order activity type transition patterns. By identifying temporal activity motifs from check-in sequences and analyzing the type distributions of motifs across various temporal topologies, potential associations between different activity types were uncovered. The main finding of this study can be summarized as follows:

- Activity type recurrence is a common phenomenon, especially when the activity sequence is longer or involves more types of activities. Recurrence tendency also exists between two separate activity processes, especially under commercial bridging modes.
- Moreover, with pure demands such as work or leisure, people tend to behave in patterns with activity recurrence, such as Star_pre, Star_pos, and Triad. However, those who tour or combine work and leisure are more likely to behave in chain-like patters.
- Different activity types emphasize distinct functions and roles in a complete activity process. Food act as the predominant central activity and often repeat in activity sequences. Activities of Community and Shopping types usually happen in the early stages, whereas Entertainment and Outdoors activities tend to take place later. Specifically, Community consistently triggers follow-up activities, while Entertainment always serves as the concluding activity.
- For the first time, we focused on the separate activity transition patterns, which allow differences in intermediate activities and fully consider the personalized behavior of people in real life. Therefore, commonalities are discovered from complex human behaviors, indicating that human behaviors separated by long intervals also influence each other.

This study focuses on daily-scale patterns, as they better align with the circadian rhythms and behavioral regularity of human activity, consistent with most motif-based human behavioral studies^23,26–28^. Extending to a multi-day scale may introduce noise and structural complexity, as cross-day transitions often involve heterogeneous contexts and less stable temporal semantics, potentially obscuring the clarity of the extracted motifs. Besides, the duration of activity types is not explicitly modeled in the definition of temporal activity motifs, as it does not affect the fundamental structure of the motifs, which aligns with many related studies^23,26–28^. However, it can serve as an additional dimension for analysis and could be considered in future directions.

Motifs make our findings more representative and robust. Moreover, they naturally represent the main features of human activity patterns, and their limited count facilitates feature construction. The features of temporal activity motifs can be linked to corresponding POIs through simple feature embedding or heterogeneous graph modeling with graph representation learning methods, enriching the behavioral context information of POIs. Although spatial information is not incorporated into the motif definitions in this study, motif-based features can be associated with geographic entities. For example, in location-based recommendation systems, by associating motifs with corresponding POIs, motif features can be extended to the spatial dimension. Motif features can also be used to characterize users’ activity transition preferences. In user-oriented location recommendations, target user preferences can be inferred and supplemented based on the preferences of users with similar motif features, thereby improving the model’s performance.

By analyzing people’s activity transition patterns, we provide some suggestions for urban planning. First, centralizing the layout of commercial activities that often appear in the same activity process is an efficient way to enhance convenience and promote consumption. Secondly, connecting or integrating indoor and outdoor activity venues can help residents achieve a balance between physical and mental well-being. Last, customizing services for specific groups such as businesspeople and tourists can improve efficiency and experiences while enhancing the city’s attractiveness, as seen in examples like transit-oriented development and tourist attractions.

Urban environments are constantly evolving. Particularly, the COVID-19 pandemic has affected human mobility, transportation modes, and activity types. During the pandemic, human mobility and public transportation sharply decreased, while active modes such as cycling and walking increased^31,32^. People reduced travel to shopping malls and tourist attractions, whereas new towns and leisure venues remained hot spots^33^. In the post-pandemic era, public transportation has steadily recovered^32^. Most activity indicators have returned to or surpassed pre-pandemic levels, while out-of-home dwell time and spatial entropy have not fully recovered, suggesting these metrics are undergoing recalibration^34^. Meanwhile, the share of work-related trips has decreased, while leisure and shopping trips have increased, reflecting a shift toward activities beneficial to the mental health^35^. Although people’s lifestyles were disrupted in the short term, they exhibit strong stability and resilience. While our dataset is relatively outdated, the analysis remains applicable, especially for activity patterns with structural regularities and long-term stability. However, changes in dwell time and non-work travel suggest that urban activity structures are evolving. Our method is applicable for newer datasets and could be used to explore post-pandemic changes in activity types in future studies.

### Limitations

There are some limitations of this study. First, the experiments were conducted solely on the Gowalla dataset. Nonetheless, the proposed method is applicable to a wide range of datasets that can be transformed into sequences of activity types, and the number of activity types can be flexibly adjusted within the algorithm. Second, we assume that the check-in data can reflect user activity type sequence. But in fact, the check-in data is affected by users’ preferences and habits, which may lead to discrepancies between our samples and the actual activity type sequences. When people use social media, they are often motivated to share their lives or gain social recognition, so they tend to check in at popular places such as tourist attractions, internet-famous shops, gyms, or cafes, and rarely check in at home, hospitals, etc. for privacy reasons. Furthermore, check-in data only represents those people who like to check in on location-based social networks. Other groups will be ignored, for example, elderly people who do not use the Internet or people who are not used to checking in. Third, our study does not focus on the continuation of the same type of activities. However, it’s common for people to continuously carry out the same activities in real life. In order to make our method better assist the recommendation system, reflexive structure can be added to temporal activity motifs, so that we can have a more comprehensive understanding of the transition and maintenance patterns of activity types.

### Future directions

Future research could further investigate the temporal and spatial distribution characteristics of activity transitions, including the analysis of their duration. Check-in data with a longer time span, along with socio-economic data, can be integrated to explore the long-term evolution of human activity patterns and uncover associations with various factors. In particular, future research could incorporate post-pandemic data to investigate the long-term impacts of the pandemic on people’s lifestyle. Besides, in terms of applications, motif features can further be applied to downstream tasks such as user profiling, user clustering, business site selection, recommendation systems, and other location-based services.

## Conclusion

In this study, a new tool called temporal activity motif is proposed to analyze activity type transitions in human behavior. We provided evidence of activity type recurrence, which tends to occur with longer activity processes and simpler travel demands. We also found that various activity types play different roles in activity processes. Furthermore, we innovatively explored non-sequential motifs, revealing the co-occurrence relationships between two activity processes. These findings provide theoretical references and practical inspiration for optimizing recommendation system and urban planning, ultimately enhancing user experience and urban development efficiency.

## Methods

**Fig. 8:**
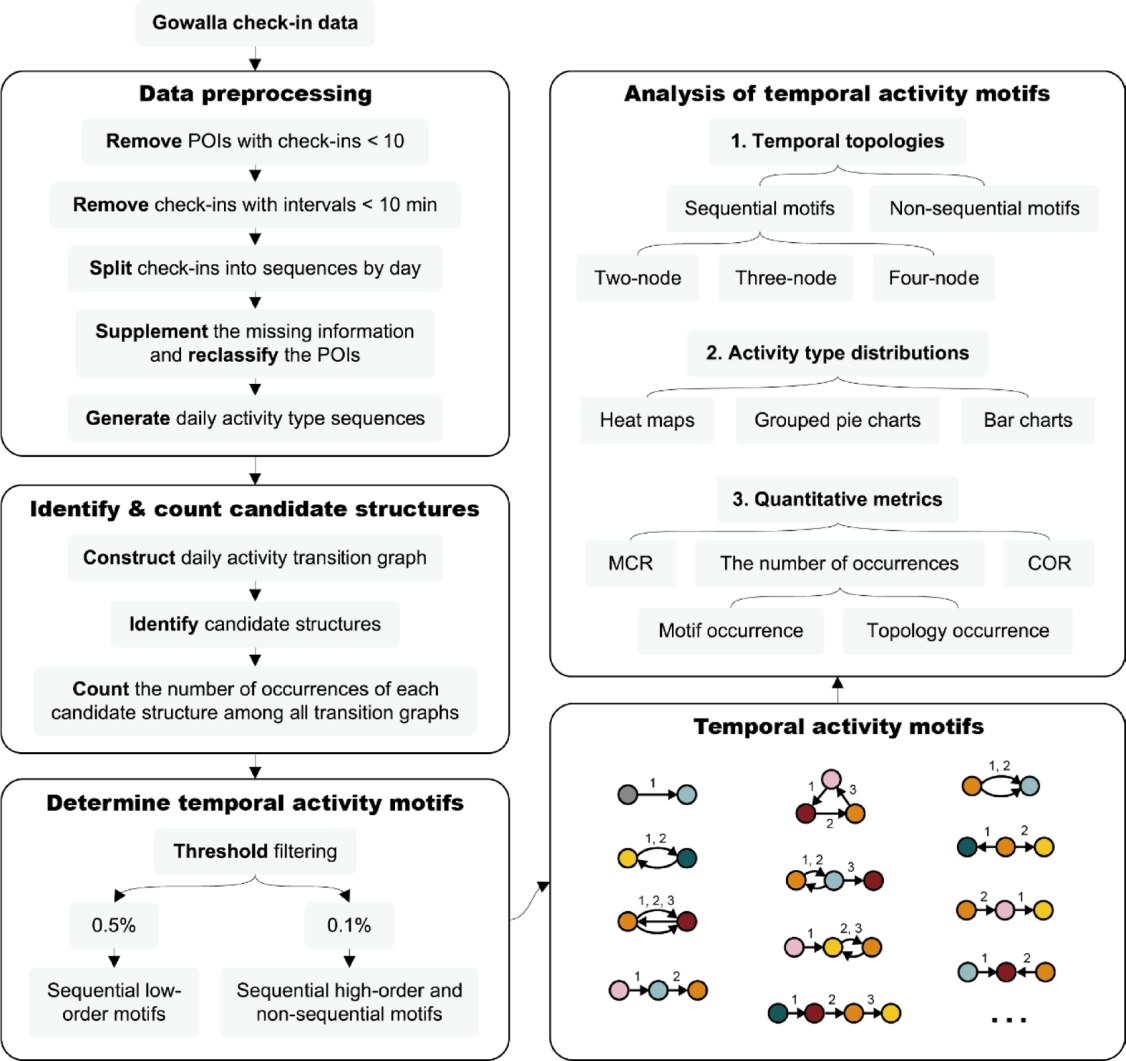
Methodological framework of the study.

The overall methodological framework adopted in this study is illustrated in Fig. 8, which outlines the process of identifying and analyzing temporal activity motifs from location-based check-in data. First, the check-in data was filtered and segmented into daily sequences of activity types. Second, these sequences were transformed into daily activity transition graphs, from which various candidate structures were identified and their occurrences were counted. Third, temporal activity motifs were determined based on two predefined threshold criteria. Finally, in the analysis phase, the identified motifs were visualized in terms of their temporal topologies and activity type distributions, and quantitative metrics such as MCR and COR were applied to support the analysis.

Data preprocessing, analysis, and visualization were carried out using Python 3.9.19, with key libraries such as Pandas 1.4.2, NumPy 1.21.5, and Matplotlib 3.8.4. For motif identification and counting, C + + 14 was employed, with the program built upon the graph structure class and temporal motif identification methods defined in an existing open-source framework (https://github.com/snap-stanford/snap)^30^.

### Data

The check-in data we used is sourced from Gowalla (https://www.gowalla.com/). We extracted and utilized user check-in data from New York-Newark-Jersey City, NY-NJ-PA, a metropolitan statistical area that includes parts of New York, New Jersey, and Pennsylvania, from March 2009 to July 2011. The raw check-in data includes 788,575 check-ins generated by 20,944 users across 50,790 POIs. In the original dataset, each row represents a check-in record, and each column denotes an attribute of the check-in, including user ID, POI ID, geographic coordinates, and datetime. Additionally, the Gowalla website provides POI category IDs and a hierarchical classification system, facilitating the inference of activity types.

### Data preprocessing

User uncertainty or system errors may lead to anomalies in the check-in data, such as excessively frequent consecutive check-ins or invalid check-in locations. Following the practices of earlier researchers^36–39^, we removed POIs with fewer than 10 check-ins and eliminated check-ins with intervals shorter than 10 min. After cleaning, 434,590 check-in records remained.

In the classification system provided by Gowalla, the first-level categories of POIs include community, entertainment, food, nightlife, outdoors, shopping, and travel, with a total of 128 second-level categories (https://www.yongliu.org/datasets/). However, this classification does not directly map to common types of user activity. Moreover, Gowalla’s information is incomplete: some POIs subcategories are not assigned to any first-level category, and some POIs lack category details. Therefore, we manually supplemented the missing information and refined the multi-level classification system. New first-level categories directly correspond to seven typical types of user activities: Residence, Community, Entertainment, Food, Outdoors, Shopping, and Travel. We categorized each check-in record into one of these activity types and labeled them, to facilitate subsequent data processing and analysis.

We divided the check-in data into 24 time periods of a day based on the timestamps and found that check-ins are least likely to occur between 4:00 and 5:00 AM, followed by 5:00 to 6:00 AM. Accordingly, we used 5:00 AM as the time boundary to segment the check-in data into 225,248 daily activity type sequences. Since we are only interested in transitions between activity types, consecutive identical types in a sequence were merged. Additionally, sequences containing only one activity were excluded from the study. After preprocessing, a total of 80,118 daily sequences from 11,723 users were suitable for analysis. The length distribution of these sequences is imbalanced. Less than half sequences (35,421) from 7,424 users have length greater than or equal to 3, while only 17,125 sequences from 4,725 users have length greater than or equal to 4, which may contain complex behaviors.

According to the activity type labels on daily sequences, each activity type transition generates a directed edge with timestamp, ultimately constructing a temporal directed graph (also called daily activity transition graph) where nodes represent activity types. To identify temporal activity motifs, we first identify and count the occurrences of candidate structures in all daily activity transition graphs and then filter out high-frequency structures as motifs based on predefined thresholds.

### Definition of Temporal activity motif

In the field of complex networks, motifs refer to subgraph structures that frequently appear in networks^22^. Schneider et al. drew an analogy to complex network motifs and proposed location-based individual mobility motifs to analyze human movement patterns^23^. They transformed user daily movement sequences into directed graphs, grouped and counted them based on topological structures, and termed the frequently occurring topologies as individual mobility motifs. Cao et al. defined activity-based motifs by additionally considering node type^26^, while Lei et al. introduced temporal motifs by incorporating temporal attributes of directed edges^28^.

We propose a novel structure called temporal activity motif and provide its definition. Temporal activity motifs, as high-frequency patterns in datasets, summarize a series of representative and primary user behaviors, thereby reflecting the prevalent activity transition patterns in the data. In fact, many low-frequency patterns also exist in the sample, but they are often subject to randomness and noise. By limiting the analysis to high-frequency motifs, we ensure robust and accurate results, minimizing disruptions from accidental factors. Temporal activity motifs are directed graphs, where nodes represent activity types and edges denote transitions between them, with timestamps on edges indicating the sequence of transitions. When identifying motifs, the following requirements need to be considered:

- More than one temporal activity motif can be identified from a single user daily activity sequence. This is because we aim to identify typical local activity patterns (maybe more than one) rather than the global pattern (only one, summarizing the entire day). Different local patterns can reflect user activity characteristics from different perspectives.
- Temporally discontinuous edges are allowed to form a motif. For example, in the sequence “Residence → Community → Outdoors → Community → Residence,” we can ignore the edges connected to “Outdoors” and identify the pattern “Residence → Community → Residence.” By omitting less significant intermediate activities and retaining the main activity process, we can better identify typical activity patterns.
- Topologically discontinuous edges are also allowed to form a motif, referred to as non-sequential motifs. For instance, in the sequence “Residence → Community → … → Food → Entertainment,” we can identify the pattern “Residence → Community, Food → Entertainment.” This two-segment pattern emphasizes the co-occurrence of the two activity processes, with other activities occurring between them receiving less attention.

To uncover daily human activity patterns, we segmented activity sequences by day before identifying temporal activity motifs. Unlike sliding time windows, this approach aligns with circadian activity cycles, which better suits our research goal of capturing daily activity transitions. As a results, all identified motifs are implicitly constrained by a 24-hour temporal boundary, ensuring they represent not only structural regularities, but also time-bounded patterns reflective of users’ daily routines. Besides, during preprocessing, check-ins with very short durations were removed, which means that all transitions in the motifs are considered substantively meaningful. Although activity transitions may vary in interval times, each reflects a certain type of need or motivation in people’s daily life. Consistent with many related studies^23,26–28^, duration of activity types is not included in our motif definition.

Spatial information is not included in our motif definition, as temporal activity motifs are designed to capture transitions between activity types, which serve as semantic abstractions of user behavior. Although activity types can be linked to specific POIs, they are not inherently spatial entities. Our focus, therefore, is on uncovering higher-order patterns in activity transitions, rather than modeling location-based dynamics.

### Identifying and counting candidate structures

To meet the three requirements mentioned in the previous section, we must identify all candidate structures in every daily transition activity graph and then count the number of graphs containing each candidate structure. This calls for a novel approach to identify multiple temporal typed structures from temporal directed graphs with node types. Existing methods for temporal motif identification and counting are divided into two categories: one is enumeration methods based on subgraph isomorphism^40–42^, which can exhaustively list all subgraphs in a network but incurs exponential time complexity as the number of nodes increases. Therefore, we opted for the other category, counter-based methods. This category of methods, first proposed by Paranjape et al.^30^, decomposes the counting problem of high-order motifs into counting problems of low-order motifs by maintaining multiple counters to store intermediate results. It relies on the design of counters based on predefined motif structures and achieves linear complexity for motif structures with up to three nodes and three edges. On this basis, researchers have attempted to identify higher-order motifs, incorporate node attributes, or design faster algorithms^43,44^. Unfortunately, none of these methods fully meet the needs of our study.

Therefore, we modified the method proposed by Paranjape et al. On one hand, we expanded the dimensionality of the counters to count different node type combinations separately, enabling the identification of motifs with node type labels. On the other hand, we designed new counter combinations to identify motifs with up to four nodes and four edges, allowing for the recognition of more complex topologies than the original method. Using this approach, we identified a total of 96,423 temporal typed candidate structures. We then calculated the number of daily activity transition graphs containing each candidate structure as its frequency in the dataset, to determine whether it qualifies as a temporal activity motif, which will be elaborated in the next section.

### Determining Temporal activity motifs

Temporal activity motifs are determined based on threshold criterions. Candidate structures with frequencies above the threshold are considered frequently occurring patterns and thus classified as motifs. Before setting the threshold, we observed that the distribution of daily activity sequence lengths in the sample is highly imbalanced. Among the 11,723 users, only 4,725 have sequences with lengths greater than or equal to 4, which are eligible to contain high-order motifs with three or more edges. Usually, these users spend more time on social media, related to higher engagement and more intricate behavior. The remaining 6,998 users have sequences with lengths of three or fewer, representing at most two activity type transitions and generally indicating lower engagement and simpler behaviors. Among the 80,118 sequences, sequential motifs with two or fewer edges are more frequently observed. Sequential high-order motifs with more than two edges and non-sequential motifs are only possible to occur in the 17,125 sequences with lengths greater than three. If the same threshold were applied to all motif types, higher-order patterns are likely to be overlooked.

Therefore, according to previous research^23,26,27^, we set two distinct thresholds: 0.1% and 0.5% of the total sample size (80,118), corresponding to 80 and 400 respectively. For sequential low-order motifs, the threshold is set at 400, allowing us to summarize simple but prevalent transition patterns from the entire dataset. For sequential high-order motifs and non-sequential motifs, the threshold is set at 80, facilitating the exploration of potential complex patterns within the core users. As a result, only 383 candidate structures are identified as motifs.

We conducted a sensitivity experiment on the selection of frequency thresholds by testing three settings: (0.08%, 0.4%), (0.1%, 0.5%), and (0.12%, 0.6%). We compared the number of occurrences and the number of motif types identified for each temporal topology (see Supplementary Fig. S1 online). The results show that the number of occurrences in dataset is largely insensitive to threshold variation, while the number of motif types decreases more noticeably as the threshold increases. Nevertheless, the overall trend remains consistent, suggesting that the analysis is robust to moderate changes in threshold values.

### Quantitative analysis metrics

The number of occurrences refers to the total count of instances where motifs or topologies appear within daily activity sequences, which serves as the basis of visualization and quantitative analysis. Specifically, motif occurrence refers to the count of instances where a specific motif appears. Topology occurrence, on the other hand, refers to the count of instances where any motif belonging to a specific temporal topology or group of topologies (comprising multiple motifs) appears.

Motif Coverage Ratio (MCR) is defined to measure the extent to which motifs belonging to specific topologies explain the dataset, mitigating bias from the imbalanced distribution of sequence lengths. The calculation formula is provided below.

$$\:\text{M}\text{C}\text{R}=\frac{{N}_{c}}{{N}_{a}}$$

For motifs with specific topology (or grouped topologies), the numerator $\:{N}_{c}$ represents the number of daily activity sequences in which they occur, i.e., the number of their occurrences in the dataset. Meanwhile, the denominator $\:{N}_{a}$ represents the number of daily activity sequences structurally allow to include them, ensuring that the ratio is calculated within a reasonable range (e.g., for three-edge motifs, the denominator is the number of sequences with lengths greater than or equal to 4).

Cumulative Occurrence Ratio (COR) is defined to quantify the relative importance of specific motifs within a given temporal topology. The calculation formula is provided below.

$$\:\text{C}\text{O}\text{R}=\frac{{C}_{s}}{{C}_{a}}$$

The numerator $\:{C}_{s}$ represents the cumulative number of occurrences of specific motifs, i.e., the sum of their respective motif occurrence. The denominator $\:{C}_{a}$ represents the total cumulative occurrences of all motifs within the given temporal topology. During the analysis, this metric is used to evaluate the importance and involvement of motifs with specific semantics.

## Supplementary Information

Below is the link to the electronic supplementary material.

Supplementary Material 1

## Data Availability

The datasets generated and/or analyzed during the current study are available in the Zenodo repository, https://doi.org/10.5281/zenodo.15067023.
