# Supplementary material for: Discovering activity transition patterns in social media check-in behavior via temporal activity motifs
Source: Sci Rep. 2025 Aug 8;15:29030. doi: 10.1038/s41598-025-14843-x (PMC12334585; doi:10.1038/s41598-025-14843-x)
Supplement: Supplementary file 1 — Supplementary Material 1 [file 41598_2025_14843_MOESM1_ESM.pdf]

# Discovering activity transition patterns in social media check-in behavior via temporal activity motifs

**Rui Zhao[1] & Yong Gao[1]***

[1]Institute of Remote Sensing and Geographic Information Systems, School of Earth and Space Sciences, Peking University, Beijing, China.
*email: gaoyong@pku.edu.cn

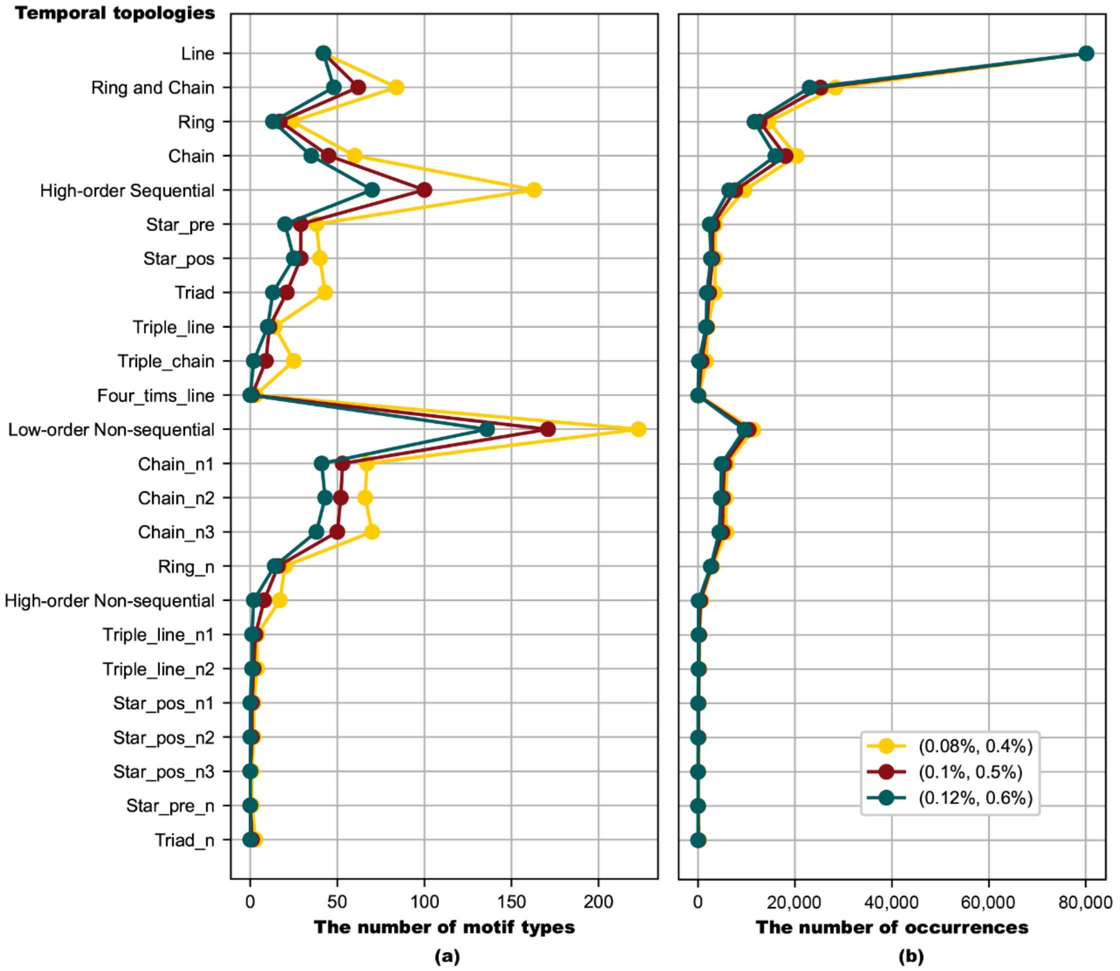


Supplementary Figure S1. Sensitivity analysis under three frequency threshold settings. The figure shows the number of motif types (a) and the number of occurrences in dataset (b) of each temporal topological structure under different threshold settings. Specifically, yellow lines represent the (0.08%, 0.4%) threshold setting, red lines represent (0.1%, 0.5%), and green lines represent (0.12%, 0.6%).
